# Self-organisation of small-world networks by adaptive rewiring in response to graph diffusion

**DOI:** 10.1038/s41598-017-12589-9

**Published:** 2017-10-13

**Authors:** Nicholas Jarman, Erik Steur, Chris Trengove, Ivan Y. Tyukin, Cees van Leeuwen

**Affiliations:** 1 Laboratory for Perceptual Dynamics, Faculty of Psychology and Educational Sciences, KU Leuven, Tiensestraat 102, B-3000 Leuven, Belgium; 2 Department of Mathematics, University of Leicester, Leicester, United Kingdom; 3 Institute for Complex Molecular Systems, Eindhoven University of Technology, Eindhoven, The Netherlands; 4 Department of Mechanical Engineering, Eindhoven University of Technology, Eindhoven, The Netherlands; 5 Saint-Petersburg State Electrotechnical University, Saint-Petersburg, Russian Federation; 6 Center for Cognitive Science, Kaiserslautern University of Technology, Kaiserslautern, Germany

## Abstract

Complex networks emerging in natural and human-made systems tend to assume small-world structure. Is there a common mechanism underlying their self-organisation? Our computational simulations show that network diffusion (traffic flow or information transfer) steers network evolution towards emergence of complex network structures. The emergence is effectuated through adaptive rewiring: progressive adaptation of structure to use, creating short-cuts where network diffusion is intensive while annihilating underused connections. With adaptive rewiring as the engine of universal small-worldness, overall diffusion rate tunes the systems’ adaptation, biasing local or global connectivity patterns. Whereas the former leads to modularity, the latter provides a preferential attachment regime. As the latter sets in, the resulting small-world structures undergo a critical shift from modular (decentralised) to centralised ones. At the transition point, network structure is hierarchical, balancing modularity and centrality - a characteristic feature found in, for instance, the human brain.

## Introduction

Complex network structures emerge in protein^1^ and ecological networks^2^, social networks^3^, the mammalian brain^4–6^, and the World Wide Web^7^. All these self-organising systems tend to assume small–world network (SWN) structure. SWNs may represent an optimum in that they uniquely combine the advantageous properties of clustering and connectedness that characterise, respectively, regular and random networks^8^. Optimality would explain the ubiquity of SWN structure; it does not inform us, however, whether the processes leading to it have anything in common. Here we will consider whether a single mechanism exists that has SWN structure as a universal outcome of self-organisation.

In the classic Watts and Strogatz algorithm^9^, a SWN is obtained by randomly rewiring a certain proportion of edges of an initially regular network. Thereby the network largely maintains the regular clustering, while the rewiring creates shortcuts that enhance the networks connectedness. As it shows how these properties are reconciled in a very basic manner, the Watts-Strogatz rewiring algorithm has a justifiable claim to universality. However, the rewiring compromises existing order than to rather develop over time and maintain an adaptive process. Therefore the algorithm is not easily fitted to self-organising systems.

In self-organising systems, we propose, network structure adapts to use - the way pedestrians define walkways in parks. Accordingly, we consider the effect of adaptive rewiring: creating shortcuts where network diffusion (traffic flow or information transfer) is intensive while annihilating underused connections. This study generalises previous work on adaptive rewiring^10–14^. While these studies have shown that SWN robustly emerge through rewiring according to the ongoing dynamics on the network, the claim to universality has been frustrated by need to explicitly specify the dynamics. Here we take a more general approach and replace explicit dynamics with an abstract representation of network diffusion. Heat kernels^15^ capture network-specific interaction between vertices and as such they are, for the purpose of this article, a generic model of network diffusion.

We study how initially random networks evolve into complex structures in response to adaptive rewiring. Rewiring is performed in adaptation to network diffusion, as represented by the heat kernel. We systematically consider different proportions of adaptive and random rewirings. In contrast with the random rewirings in the Watts-Strogatz algorithm, here, they have the function of perturbing possible equilibrium network states, akin to the Boltzmann machine^16^. In this sense, the perturbed system can be regarded as an open system according to the criteria of thermodynamics.

In adaptive networks, changes to the structure generally occur at a slower rate than the network dynamics. Here, the proportion of these two rates is expressed by what we call the diffusion rate (the elapsed forward time in the network diffusion process before changes in the network structure). Low diffusion rates bias adaptive rewiring to local connectivity structures; high diffusion rates to global structures. In the latter case adaptive rewiring approaches a process of preferential attachment^17,18^.

We will show that with progressive adaptive rewiring, SWNs always emerge from initially random networks for all nonzero diffusion rates and for almost any proportion of adaptive rewirings. Depending on diffusion rate, modular or centralised SWN structures emerge. Moreover, at the critical point of phase transition, there exists a network structure in which the two opposing properties of modularity and centrality are balanced. This characteristic is observed, for instance, in the human brain^19–21^. We call such a structure hierarchical. In sum, adaptation to network diffusion represents a universal mechanism for the self–organisation of a family of SWNs, including modular, centralised, and hierarchical ones.

## Results

For each pair (*τ*, *p*), where *τ* is a heat kernel parameter, and *p* is the rewiring probability, 100 independently evolved networks are obtained by our adaptive rewiring algorithm (see Methods). Resulting networks are described according to measures of small-world structure, modularity, and centrality. Where appropriate, the average of such measures is taken.

### Small-World Structure

The *small-worldness* index *S* provides a canonical measure of the degree to which a network is small-world. Here, we take a slightly modified version, in which the normalised clustering coefficient (*C*) is multiplied by the normalised global efficiency (*E*)^8^, such that *S* = (*C*/*C*_*r*_) × (*E*/*E*_*r*_), where *C*_*r*_ and *E*_*r*_ are measures of *C* and *E* for an equivalent Erdös-Rényi (ER) random network^22^. In doing so, *S* is also defined on disconnected networks.

For random networks, *S* ≈ 1 and so the greater the (positive) deviation of *S* from one, the greater the degree of small-worldness. For comparison, we include the average small-worldness values for networks constructed by the Watts-Strogatz algorithm (100 independently constructed networks for each *p* = 0, 1/500, 2/500, …, 1).

**Figure 1:**
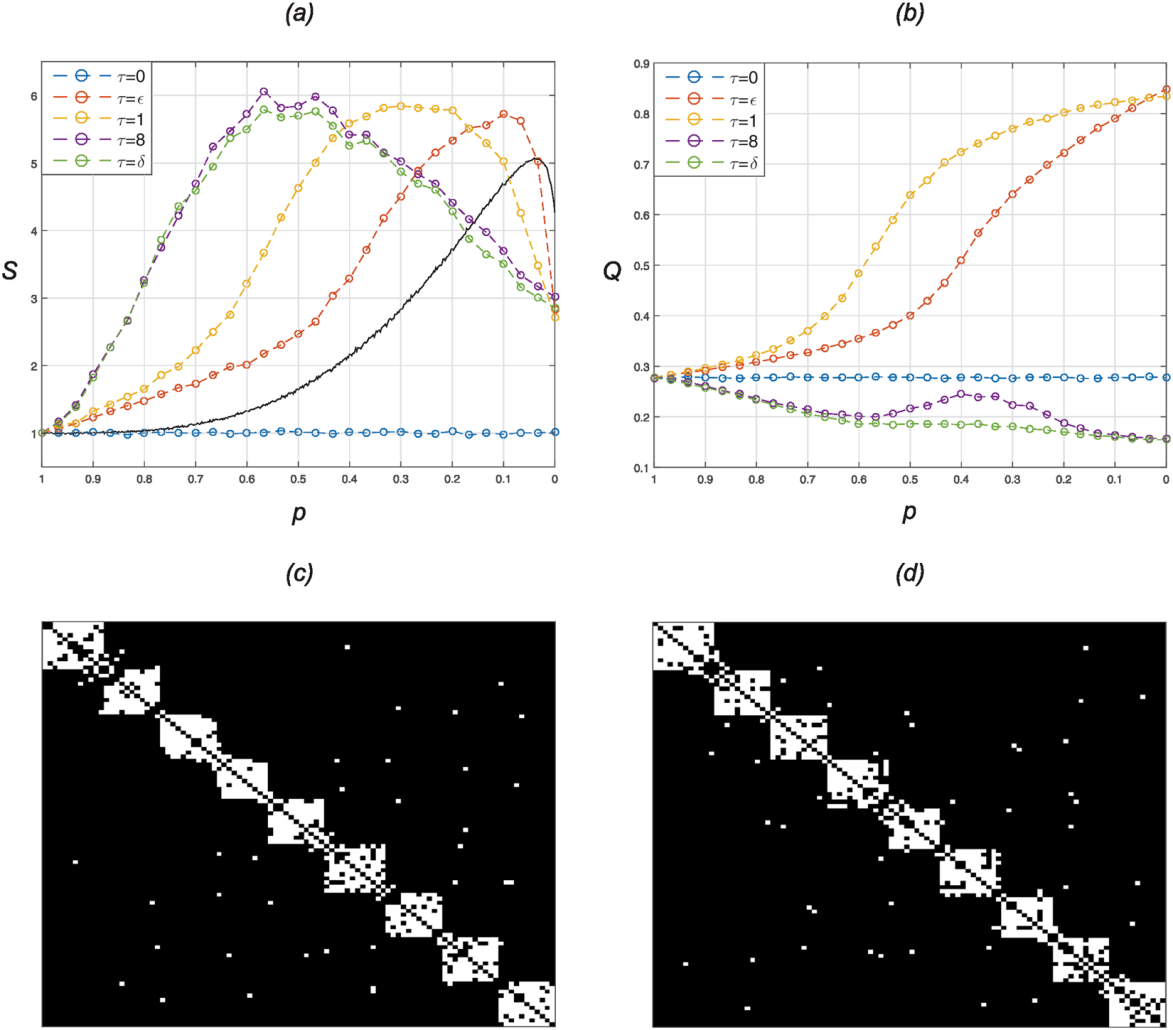
(**a**) Depicts the small-world index *S* as a function of decreasing random rewiring probability *p* ∈ {0, 1/30, …, 29/30, 1}: Coloured lines indicate values of heat kernel parameter $\tau \in \mathrm{\{0,}\,\varepsilon ,\,\mathrm{1,}\,\mathrm{8,}\,\delta \}$,  black line indicates the Watts-Strogatz algorithm with random rewiring probability $p\in \mathrm{\{0,}\,\mathrm{1/500,}\,\ldots ,\,\mathrm{499/500,}\,\mathrm{1\}}$. (**b**) Depicts the average modularity *Q* as a function of decreasing random rewiring probability $p\in \mathrm{\{0,}\,\mathrm{1/30,}\,\ldots ,\,\mathrm{29/30,}\,\mathrm{1\}}$: Coloured lines indicate values of heat kernel parameter $\tau \in \mathrm{\{0,}\,\varepsilon ,\,\mathrm{1,}\,\mathrm{8,}\,\delta \}$. (**c,d**) Single trial. Example modular SWN. Adjacency matrices mapped to an *n*-by-*n* grid where rows (and columns) represent vertices and white indicates the existence of an edge. Rows and columns of adjacency matrices have been permuted to visualise the modules, in accordance with^28^. (**c**) $(\tau ,\,p)\,=\,(\varepsilon ,\,\mathrm{0.1)}$; (**d**) $(\tau ,\,p)\,=\,\mathrm{(1,}\,\mathrm{0.3)}$.

In Fig. 1a we observe the average small-worldness index *S* as a function of random rewiring probability *p*. Unless stated otherwise, we consider networks arising from adaptive rewiring for random rewiring probability *p *∈ {0, 1/30, …, 29/30, 1}. A striking result is that SWN emergence is observed for all sample values of *τ* nonzero, no matter how small or large. Moreover, for all nonzero *τ* that have been explored a greater maximum small-worldness is achieved than with the Watts-Strogatz algorithm.

The degree of network adaptation to network diffusion, 1 − *p*, for which maximum small-worldness is obtained depends on the rate of diffusion *τ*. The values of *τ* are taken from the set {0, 10^−15^, 1, 8, 10^15^}. For the sake of convenience, we denote 10^−15^ = *ε*, 10^15^ = *δ*. For *τ* = 0, adaptive rewiring is a random process; emergent networks thus reflect those of the initial ER ones. For *τ* ∈ {8, *δ*}, the heat kernel reflects the degree distribution, and thus adaptive rewiring approaches a process of preferential attachment (see Methods). For maximum small-worldness, adaptive rewiring in response to local diffusion, for *τ* ∈ {*ε*, 1}, requires small *p*, i.e. small degree of random rewiring, while more global diffusion - preferential attachment -, for *τ* ∈ {8, *δ*}, requires larger *p*.

Interestingly, in Supplementary Fig. S1, for *τ* ∈ {8, *δ*} and *p* large, the network achieves an even greater efficiency than an equivalent random ER network, i.e. it is more well integrated.

### Modular Structure

The modularity index *Q* is an optimised statistic of network partitioning into non-overlapping communities. The value *Q* is calculated as the proportion of intra-modular connections minus the expected proportion of inter-modular connections for an equivalent ER random network under the same community structure^23,24^.

In Fig. 1b we observe the average modularity index *Q* as a function of random rewiring probability *p*. We observe that modularity can be controlled by choice of pair (*τ*, *p*). This is discussed in further detail in the section *Critical Network Structure*. For *τ* ∈ {*ε*, 1} networks emerge with near-maximal degrees of modularity as *p* → 0. On the other hand, for *τ* ∈ {8, *δ*} and over all sampled *p* ∈ [0, 1] emergent networks posses no community structure, i.e. modularity is essentially switched off. In fact, we see a lesser degree of modularity than in an equivalent random ER network.

In Fig. 1c,d we present the adjacency matrices, permuted to visualise the modules, from randomly sampled networks resulting from single independent trials of the algorithm with pairs (*τ*, *p*), where *p* = *p*(*τ*) is chosen dependent on *τ* so that *S* is at maximum. In both Fig. 1c,d where (*τ*, *p*) = (*ε*, 0.1) and (*τ*, *p*) = (1, 0.3), respectively, emergent modules are relatively uniform with a dense intra-connectivity and sparse inter-connectivity.

### Centralised Structure

Properties of centrality are characterised using the measures of PageRank, the degree, assortativity, and maximised coreness statistic. The latter three network measures are evaluated for pairs (*τ*, *p*) where *p* is chosen dependent on *τ* such that *S* is at maximum.

The *PageRank centrality* vector, a variant of eigenvector centrality, is defined as the stationary distribution achieved by instantiating a Markov chain on a network^25,26^, i.e., the probability distribution that a random walker is located at a given vertex. PageRank centrality takes into account global communication patterns, mediated by longer path lengths and patterns of convergence and divergence, whereas some of the more common centrality measures, such as closeness and betweenness centrality, do not^27^. We denote as *π* the (normalised) maximum element of the PageRank vector. We take equal initial PageRank probability, and take the damping factor (the probability of transitioning to an adjacent vertex) as 0.85^28^, i.e., the probability of random vertex hopping is 0.15. Since the PageRank vector sums to one, then its mean value is 1/*n*. For convenience we normalise *π* by this mean value.

**Figure 2:**
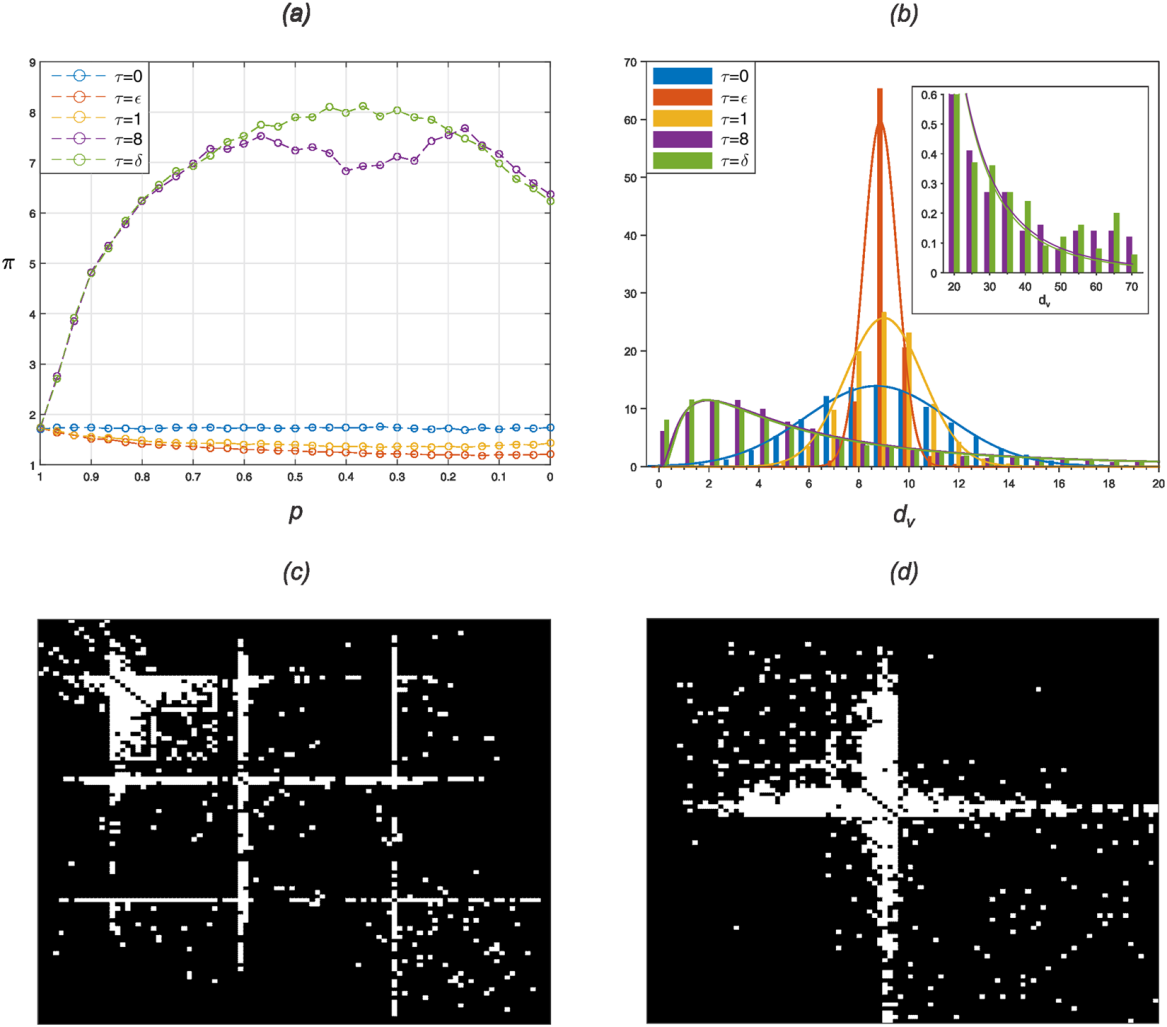
(**a**) Depicts the average *π* - maximum element of PageRank vector normalised by its mean - as a function of decreasing random rewiring probability $p\in \mathrm{\{0,}\,\mathrm{1/30,}\,\ldots ,\,\mathrm{29/30,}\,\mathrm{1\}}$: Coloured lines indicate values of heat kernel parameter $\tau \in \mathrm{\{0,}\,\varepsilon ,\,\mathrm{1,}\,\mathrm{8,}\,\delta \}$. (**b**) Depicts the bar-plot in which the height of individual bars is the average number of vertices having degree *d*_*v*_, where ${d}_{v}\le 20$. Inset bar-plot for vertex degrees *d*_*v*_, where 20 ≤ *d*_*v*_ ≤ 70. Probability density function (PDF) curves fitted to *d*_*v*_: truncated normal PDF for $\tau \in \mathrm{\{0,}\,\varepsilon ,\,\mathrm{1\}}$ and truncated and normalised lognormal PDF for $\tau \in \mathrm{\{8,}\,\delta \}$. Coloured bars (and curves) indicate values of heat kernel parameter $\tau$; for each, $p$ is chosen dependent on $\tau$ such that *S* is at maximum. (**c,d**) Single trial. Example centralised SWN. Adjacency matrices mapped to an $n$-by-$n$ grid where rows (and columns) represent vertices and white indicates the existence of an edge. Rows and columns of adjacency matrices have been permuted to visualise the modules, in accordance with^28^. (**c**) Depicts $(\tau ,\,p)\,=\,\mathrm{(8,\; 0.5667)}$; (**d**) depicts $(\tau ,\,p)=(\delta \mathrm{,\; 0.5667)}$.

In Fig. 2a we observe the average PageRank value *π* as a function of random rewiring probability *p*. As with modularity, we see that centrality can be controlled depending on the choice of *τ*. For *τ* ∈ {8, *δ*} emergent network structures exhibit values of *π* considerably (positively) far from that of the ER networks, indicating large deviations of the maximum component from the mean of the PageRank vector. Therefore, there exists at least one vertex having a greatly increased likelihood of being traversed in a random Markov chain than all others. On the other hand, for *τ* ∈ {*ε*, 1} and over all sampled *p* ∈ [0, 1], emergent networks posses no such central vertices, i.e. centrality is tuned off. In fact, we see a lesser degree of centrality than in an equivalent random ER network.

In accordance with Fig. 1b, depending on the values of the pair (*τ*, *p*), emergent networks are either modular or centralised. The phase transition of network structure is discussed in the section *Critical Network Structure*.

In Fig. 2b we present a bar-plot of networks’ *degree distribution*. The height of individual bars is the average number of vertices, over 100 independently evolved networks, having degree *d*_*v*_ where *d*_*v*_ = 1, …, 70. The degree distribution for *τ* ∈ {*ε*, 1} fits a truncated normal function, while for *τ* ∈ {8, *δ*} it fits a truncated log-normal function. Moreover, for *τ* ∈ {8, *δ*} vertices emerge having remarkably high degrees (=70).

In Fig. 2c,d we present the adjacency matrices, permuted to visualise the modules, from randomly sampled networks resulting from single independent trials of the algorithm with pairs (*τ*, *p*), where *p* = *p*(*τ*) is chosen dependent on *τ* so that the values of *S* are at maximum. In both Fig. 2c where (*τ*, *p*) = (8, 0.5667), and Fig. 2d where (*τ*, *p*) = (*δ*, 0.5667), we observe a small subset of hub vertices connecting to many peripheral vertices.

The *assortativity coefficient a* describes the “assortative mixing” of vertex degrees, i.e. the preference for high-degree vertices to attach to other high-degree vertices^29^. In Table 1 row *a*, for *τ* ∈ {8, *δ*} and *p* = *p*(*τ*) such that *S* is at maximum, a strong negative correlation indicates that vertices of a high degree typically connect to vertices of a low degree. On the other hand, for *τ* ∈ {*ε*, 1} an approximately zero correlation indicates no preference of connections between vertices of varying degrees.

**Table 1:** Column wise *τ*, *p* = *p*(*τ*) such that *S* is at maximum. Row wise: *a* assortativity coefficient; *c* maximised core-periphery statistic. Values presented are averages over trials.

*τ*	0	*ε*	1	8	*δ*
*a*	−0.0219	0.0259	0.0905	−0.4689	−0.5094
*c*	0.2482	0.0491	0.1317	0.8770	0.9066

The *maximised coreness statistic c* measures the extent to which a network may be well-partitioned into two non-overlapping groups of vertices, a core and a periphery group^23,30^. In Table 1 row *c*, for *τ* ∈ {8, *δ*} and *p* = *p*(*τ*) such that *S* is at maximum, values close to one indicate that the network may be well-partitioned into non-overlapping groups of core and peripheral vertices. On the other hand, for *τ* ∈ {*ε*, 1} values close to zero indicate no such core-periphery partition.

In sum, we note that for *τ* ∈ {8, *δ*}, and *p* = *p*(*τ*) such that *S* is at maximum, networks emerge as centralised, with a strong core, and that those core vertices connect to a high number of peripheral vertices. On the other hand, for *τ* ∈ {*ε*, 1}, and all sampled *p* ∈ [0, 1], networks exhibit none of these properties.

### Critical Network Structure

We consider the transition between modularity and centrality, and show that at the phase transition of network structure, the two seemingly opposing properties are reconciled. Properties of modularity are characterised by *Q* while properties of centrality are characterised by *π*.

**Figure 3:**
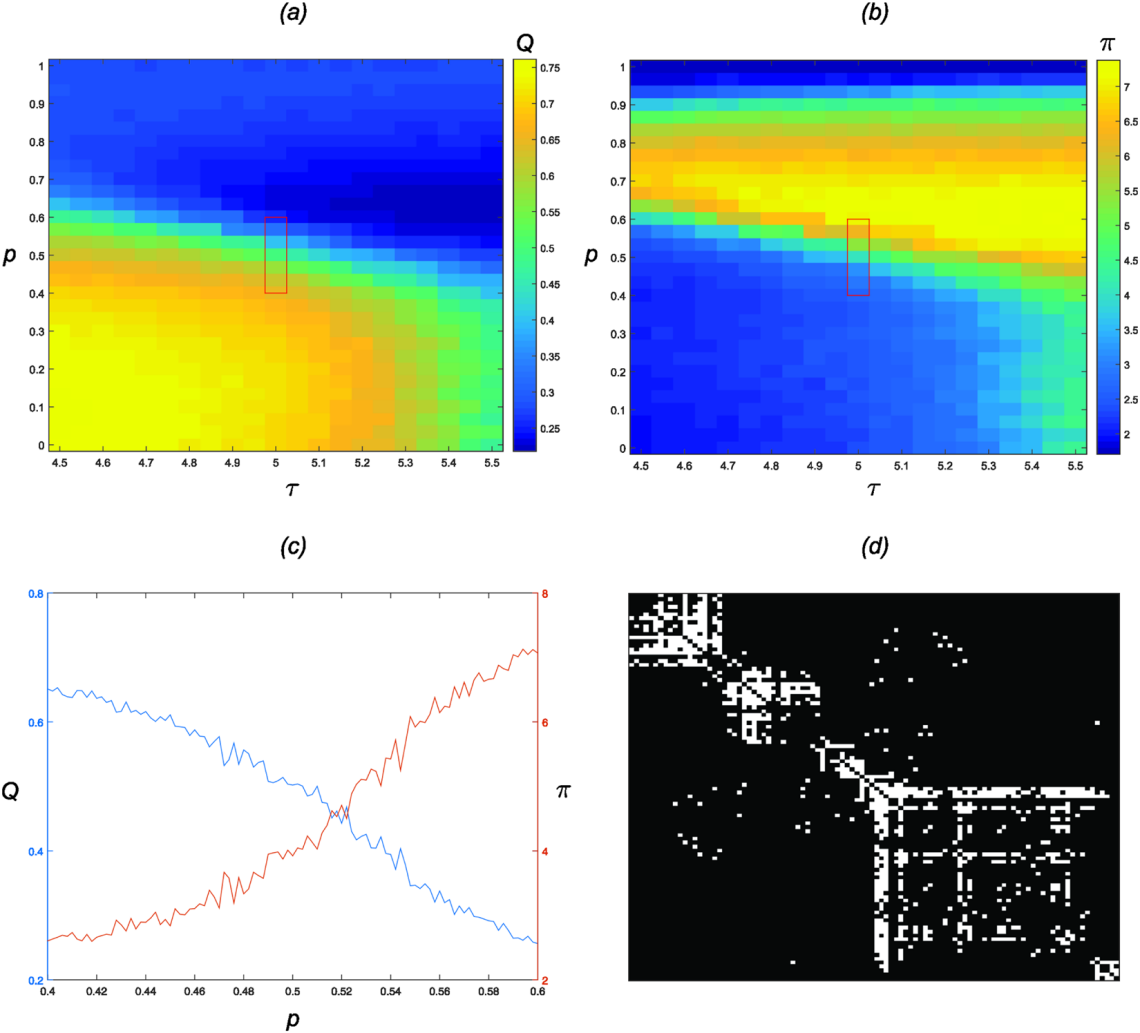
(**a,b)** In the plane $\tau \in \mathrm{\{4.50,}\,\mathrm{4.55,}\,\ldots ,\,\mathrm{5.45,}\,\mathrm{5.50\}}$ along the horizontal axis and random rewiring probability $p\in \mathrm{\{0,}\,\mathrm{1/30,}\,\ldots ,\,\mathrm{29/30,}\,\mathrm{1\}}$ along the vertical axis. (**a)** Depicts the modularity index $Q$; (**b)** Depicts *π*, the maximum element of the PageRank vector normalised by its mean value. (**c)** For $\tau \,=\,5$, along the horizontal axis random rewiring probability $p\in \mathrm{\{0.400,}\,\mathrm{0.402,}\,\ldots ,\,\mathrm{0.598,}\,\mathrm{0.600\}}$. Along the vertical axis are $Q$ the modularity index (left, blue), and $\pi$ the maximum element of PageRank vector normalised by its mean value (right, red). (**d**) Single trial. Example critical SWN. Adjacency matrix mapped to an $n$-by-$n$ grid where rows (and columns) represent vertices and white indicates the existence of an edge. Rows and columns of adjacency matrices have been permuted to visualise the modules, in accordance with^28^. Pair $(\tau ,p\mathrm{)\; =\; (5,\; 0.522)}$.

In Fig. 3a,b, for parameters *τ* ∈ {4.50, 4.55, …, 5.45, 5.50}, and *p* ∈ {0, 1/30, …, 29/30, 1}, we present *Q* (Fig. 3a), and *π* (Fig. 3b), averaged over 100 independently evolved networks. In the domain (*τ*, *p*) there is a broad region of high modularity where both *τ* and *p* are low, and a broad region of high centrality in the remainder. Where the domain of modularity ends, the domain of centrality begins; the system exhibits a critical transition from modular (decentralised) to centralised structure as a function of the pair (*τ*, *p*). The phase transition region between the two is relatively sharp with respect to both *τ* and *p*.

In Fig. 3c, we fix *τ* = 5 and take *Q* and *π* as functions of *p* ∈ {0.400, 0.402, …, 0.598, 0.600}, averaged over 100 independently evolved networks. It is clear that modularity and centrality are opposing, however, at the boundary of modularity and centrality, where they intersect at around *p* = 0.52, there is a small domain of *p* for which networks are a blend of both modular and central structure: each of *Q* and *π* are considerably large, indicating the presence of both network structures. Furthermore, the value of small-worldness for pair (*τ*, *p*) = (5, 0.522) is *S* = 5.32, indicating the network is also strongly small-world.

In Fig. 3d we present the adjacency matrix, permuted to visualise the modules, resulting from one trial of the algorithm with (*τ*, *p*) = (5, 0.522). We observe a competition between modular and centralised structure; the simultaneous existence of densely connected communities (decentralised) and a core of high degree vertices connecting to many low degree peripheral vertices (centralised). In Supplementary Fig. S2a–d we present four additional randomly sampled networks resulting from single independent trials of the algorithm with (*τ*, *p*) = (5, 0.522). These additional figures support the notion that centrality and modularity are opposing, that at the point of phase transition they are reconciled, and that this is critical, i.e. they are competitive. The adjacency matrices exhibit some degree of both centrality and modularity: emergent networks may appear as more centralised (Fig. S2a–b), or more modular (Fig. S2c), or a blend of the two (Fig. S2d).

## Conclusion and Discussion

Small-world structure offers optimal efficiency in network communication^8^; and has been shown to facilitate synchronisation in a range of oscillator networks^31–33^. Here, we studied whether a simple generic mechanism could be responsible for their emergence.

We proposed a mechanism of network self-organisation that relies on ongoing network diffusion; over time, the network is rewired adaptively, rendering it conform to the patterns of network diffusion. With some probability *p*, the adaptive rewiring process is perturbed by random rewiring. Small-world structure emerged for almost any proportion of random rewiring, moreover, networks reached higher degrees of SWN structure than those in the Watts and Strogatz algorithm^9^.

Patterns of network diffusion may be biased by local or global connectivity structures using the diffusion rate *τ*. For all (positive) nonzero diffusion rates SWN structure emerges; for small *τ*, SWNs are modular (decentralised), whereas for large *τ*, SWNs are centralised. For the latter, adaptive rewiring approaches a process of preferential attachment.

Modularity versus centrality constitutes an important dimension in the characterisation of networks in the human brain, where they play a role in terms of (structural and functional) segregation and integration^34,35^.

For intermediate values of *τ* and *p* there is a critical transition point at which network structures emerge that blend modularity and centrality. We may call these “hierarchical”^36^. Such networks are desirable for natural information processing systems like the human brain, in which a core of centralised components represents a global workspace and the decentralised modules represent autonomous client systems^19–21^. The criticality of these architectures renders them all but robust. At the level of the neuro-anatomy of the brain, it would probably involve dynamic maintenance to keep these architectures at the critical point. As a property of functional architecture, the criticality would render cognition extremely flexible, enabling rapid switching between centralised and modular processes^37^.

## Methods

Here we will provide a formal definition of network diffusion, an algorithm for adaptive rewiring, and a description of a set of computational simulations to demonstrate the role of adaptive rewiring in the generation of small-world networks. The MATLAB code of the algorithm is included in the Supplementary materials.

### Notation

In what follows we consider graphs that are undirected. A graph is an ordered pair *G* = (*V*, *E*) where *V* is the set of vertices and *E* is a subset of *V* × *V* called the edges. If *X* is a finite set, then |*X*| denotes its cardinality. The total number of vertices and edges in the graph are |*V*| = *n* and |*E*| = *m*, respectively. Two vertices *u*, *v* ∈ *V* are called *adjacent* if (*u*, *v*) ∈ *E*. For an *n*_1_ × *n*_2_ matrix *B*, *B*_*ij*_ corresponds to the entry in the *i*-th row and *j*-th column, where $i,j\,\in \,{{\mathbb{N}}}_{0}$, and $i\le {n}_{1}$, $j\le {n}_{2}$. The *adjacency matrix* of a simple graph $G$ is a square $n\times n$ matrix $A$ with entries ${a}_{uv}\,=\,1\,{\rm{if}}\,(u,v)\in E$, and ${a}_{uv}\,=\,0$ otherwise. For undirected graphs $A$ is symmetric. It is typically the case that ${a}_{uu}\,=\,0$, i.e. no self–loops. The *degree* ${d}_{v}$ of a vertex $v$ is the number of vertices adjacent to vertex $v$: ${d}_{v}={\sum }_{u\in V,u\ne v}{a}_{vu}$. The matrix $D$ is the diagonal matrix of degrees having entries ${D}_{uv}={d}_{u}$ if $u=v$ and 0 otherwise. For a given set of $n$ vertices *V* the complete graph is denoted as *K*_*n*_ and its edge set is denoted as ${E}^{{K}_{n}}$. The compliment of an edge set *E*, denoted as ${E}^{c}$, is ${E}^{c}={E}^{{K}_{n}}\backslash E$.

### Network Diffusion

The Laplacian matrix of the graph $G$ is $L=D-A$. The normalised Laplacian matrix, ${\mathcal L}$, is regarded as more appropriate for dealing with irregular graphs,

1 $$ {\mathcal L} ={D}^{-\mathrm{1/2}}L{D}^{-\mathrm{1/2}}=I-{D}^{-\mathrm{1/2}}A{D}^{-\mathrm{1/2}}$$

with the convention that ${D}_{uu}^{-1}\,=\,0$ for ${d}_{u}\,=\,0$.

All eigenvalues of ${\mathcal L}$ are real (since ${\mathcal L}$ is symmetric real) and confined to the interval $\mathrm{[0,}\,\mathrm{2]}$, in accordance with Gershgorin circle theorem^38^, and relate well to other graph invariants, such as random walks (or Markov chains), in a way that the eigenvalues of the Laplacian matrix $L$ and adjacency matrices often fail to do^39^. Let ${\lambda }_{i}$ denote the eigenvalues of ${\mathcal L}$ with eigenvectors ${v}_{i}$, and ${\omega }_{i}$ the eigenvalues of the corresponding Markov process $M$ with eigenvectors ${u}_{i}$. Then, ${\lambda }_{i}\mathrm{=1}-{\omega }_{i}$ and ${v}_{i}={D}^{\mathrm{1/2}}{u}_{i}$.

Whereas ${\mathcal L}$ incorporates information of the local connectivity of vertices, the introduction of a graph kernel provides a global connectivity metric. Physical processes such as diffusion suggest a natural way of constructing a kernel from such local information^15^.

Network diffusion is formally represented by the Exponential Heat Kernel of the graph (cf. Theorem 10.11 in^39^).

#### **Definition 1**

(*Exponential heat kernel*) *Let* ${\mathcal L}$ *be the normalised Laplacian matrix for an* $n\times n$ *real symmetric matrix and* $t\ge 0$. *The exponential heat kernel of* ${\mathcal L}$, *denoted by* $h(t)$, *is the symmetric and positive definite* $n\times n$ *matrix*,

2 $$h(t)={e}^{-t {\mathcal L} }=\sum _{k\mathrm{=0}}^{\infty }\frac{{(-t)}^{k}}{k!}{ {\mathcal L} }^{k}\mathrm{.}$$

*In particular* $h\mathrm{(0)}\,=\,I$, *the identity matrix*.

The matrix exponential is a weighted sum of walks^40^. Coefficients $\frac{{(-t)}^{k}}{k!}$ in Equation (2) allow for biasing of path lengths in the construction of $h(t)$, where for small $t$ shorter paths carry greater weight and longer paths carry lesser weight, and *vice versa*. In our simulations we use the parameter $\tau =t$.

The matrix $h(t)$ as $t\to \infty$ can be expressed by the leading eigenvector associated with the zero eigenvalue of ${\mathcal L}$. Since ${\mathcal L}$ is real and symmetric, there exists an orthonormal matrix $Q$ such that ${\mathcal L} =Q{\rm{\Lambda }}{Q}^{-1}$ where $Q$ is the matrix of eigenvectors and ${\rm{\Lambda }}$ is the diagonal matrix of eigenvalues. It is easily shown that substitution of this eigendecomposition into the Taylor expansion yields $h(t)=Q{e}^{-t{\rm{\Lambda }}}{Q}^{-1}$. Let ${{\rm{\Lambda }}}_{ii}={\lambda }_{i}$ and order the eigenvalues such that $0\,=\,{\lambda }_{0}\le {\lambda }_{1}\le \cdots \le {\lambda }_{n-1}$. If $G$ is connected, then ${\mathcal L}$ has one simple zero eigenvalue ${\lambda }_{0}$. Then, the first column of $Q$ contains the leading eigenvector, denoted as $q$, associated with the zero eigenvalue ${\lambda }_{0}$. Then, $q=\frac{{D}^{1/2}{\bf{\text{1}}}}{\sqrt{{{\bf{\text{1}}}}^{{\rm{T}}}D{\bf{\text{1}}}}}$ for ${\bf{\text{1}}}$ the $n$-vector of ones, and ${lim}_{t\to \infty }h(t)=q{q}^{{\rm T}}$.

If $G$ is regular - all vertices have equal degree $d={d}_{v}$ for all $v\in V$ - then, ${\mathcal L} =\frac{1}{d}L$, and $q\in {\rm{s}}{\rm{p}}{\rm{a}}{\rm{n}}\,(1,\ldots ,1)$, hence ${lim}_{t\to {\rm{\infty }}}h(t)=\frac{1}{n}{{\bf{\text{11}}}}^{{\rm{T}}}$. However, if $G$ is irregular, then $q\in {\rm{s}}{\rm{p}}{\rm{a}}{\rm{n}}\,(\sqrt{{d}_{1}},\ldots ,\sqrt{{d}_{n}})$, thus ${lim}_{t\to {\rm{\infty }}}h(t)=\frac{1}{{\bf{\text{1}}}D{{\bf{\text{1}}}}^{{\rm{T}}}}{D}^{1/2}{{\bf{\text{1}}}}^{{\rm{T}}}{\bf{\text{1}}}{D}^{1/2}$.

The use of ${\mathcal L}$ over $L$ in construction of the heat kernel becomes apparent for $G$ irregular. Assuming $G$ is irregular, then the off-diagonal entries of $h(t)$ as $t\to \infty$ are proportional to the square root of the vertex degrees. Thus, for $t$ taken arbitrarily large, irregularities in ${\mathcal L}$ also appear in $h(t)$, i.e. information of network structure is still contained in $h(t)$. This property does not hold if we were to replace ${\mathcal L}$ with $L$ in the construction of $h(t)$. Indeed, denote the heat kernel constructed using $L$ as ${h}_{L}(t)$, then for $G$ irregular, ${lim}_{t\to {\rm{\infty }}}{h}_{L}(t)=\frac{1}{n}{{\bf{\text{11}}}}^{{\rm{T}}}$. Note also, that for $\alpha \, > \,0$ where $\alpha$ may be taken arbitrarily small, $h(\alpha )\ne I$, i.e. off-diagonal entries of $h(\alpha )$ are nonzero, and hence $h(\alpha )$ contains information of network structure. This property holds for both the use of ${\mathcal L}$ and $L$ in construction of the heat kernel.

### Adaptive rewiring algorithm

Consider an undirected graph with number of vertices $n$ and number of edges $m$. For convenience we take $m=\lfloor 2\rho n(n-\mathrm{1)}\rceil$, the nearest integer, where $\rho =\frac{log(n)}{n}$ (natural logarithm), i.e. twice the critical connection density for which a random Erdös–Rényi (ER) graph is connected with probability one^41,42^.

We consider self–organisation starting from a random network. The network is progressively rewired, with probability $p$ at random and with probability $1-p$ according to the current network diffusion. The process can be described in algorithmic form:

Step 0: Generate an undirected random graph $G$ of the Erdös–Rényi type. Begin with the graph $G=(V,E)$ such that $|V|=n$ and $|E|\,=\,0$. Select uniformly at random the pair $u,v$ from the set $\{u,v\in V|u\ne v,(u,v)\in {E}^{c}\}$ and add the (undirected) edge $(u,v)$ to the edge set $E$. Repeat until $|E|=m$.

Step 1: Select a vertex $v$ uniformly at random from all nonzero degree vertices $v\in \{u\in V|{d}_{u}\ne 0\,{\rm{and}}\,{d}_{u}\ne n-\mathrm{1\}}$.

Step 2: Delete the edge $(v,{u}_{1})$ and add the edge $(v,{u}_{2})$ where vertices ${u}_{1}$ and ${u}_{2}$ are selected by the following criteria: With probability $p$ go to 2i, otherwise go to 2ii,

i. Vertices ${u}_{1}$ and ${u}_{2}$ are selected uniformly at random from the sets ${u}_{1}\in \{u\in V|(v,u)\in E\}$ and ${u}_{2}\in \{u\in V|(v,u)\in {E}^{c}\}$.
ii. For adjacency matrix $A$ (of graph $G$), calculate the heat kernel $h(t)$ for $t=\tau$, where $\tau$ is a chosen parameter. Vertices ${u}_{1}$ and ${u}_{2}$ are chosen such that, for all $u\in V$ and $u\ne v$,

$${u}_{1}:{h}_{v{u}_{1}}(t)\le {h}_{vu}(t)\,{\rm{for}}\,{\rm{all}}\,(v,u)\in E$$

$${u}_{2}:{h}_{v{u}_{2}}(t)\ge {h}_{vu}(t)\,{\rm{for}}\,{\rm{all}}\,(v,u)\in {E}^{c}\mathrm{.}$$

where ${h}_{uv}(t)$ is the $u,v$ entry in matrix $h(t)$. In case of ties ${u}_{1},{u}_{2}$ are chosen arbitrarily.

Step 3: Repeat from Step 1 until *k* edge rewirings have been made.

All simulations were performed using MATLAB R2014. In Step 3 we take $k\,=\,4m$; simulations without upper limit on *k* show sufficient convergence after only *m* rewirings. We simulate 100 independent trials for each pair $(\tau ,p)$. In analysing the networks generated by the algorithm all measures used are provided by the *Brain Connectivity Toolbox* ^28^. Note that for $\tau \gg 1$ the heat kernel approaches the matrix $\frac{1}{{\bf{\text{1}}}D{{\bf{\text{1}}}}^{{\rm{T}}}}{D}^{1/2}{{\bf{\text{1}}}}^{{\rm{T}}}{\bf{\text{1}}}{D}^{1/2}$ and so rewiring biases toward high degree vertices, hence, adaptive rewiring approaches a process of preferential attachment^17,18^.

## Electronic supplementary material

Supplementary Info File #1
