# Supplementary material for: Self-organisation of small-world networks by adaptive rewiring in response to graph diffusion
Source: Sci Rep. 2017 Oct 13;7:13158. doi: 10.1038/s41598-017-12589-9 (PMC5640682; doi:10.1038/s41598-017-12589-9)
Supplement: Supplementary file 1 — Supplementary Info File #1 [file 41598_2017_12589_MOESM1_ESM.pdf]

Supplementary Information: Self-organisation of small-world networks by adaptive rewiring in response to graph diffusion

Nicholas Jarman,[1,2*] Erik Steur,[3] Chris Trengove,[1]
Ivan Yu Tyukin,[2,4] Cees van Leeuwen[1,5]

[1]Laboratory for Perceptual Dynamics, Faculty of Psychology and Educational Sciences
KU Leuven, Tiensestraat 102, B-3000, Leuven, Belgium
[2]Department of Mathematics, University of Leicester, UK
[3]Institute for Complex Molecular Systems, Department of Mechanical Engineering,
Eindhoven University of Technology, The Netherlands
[4]Saint-Petersburg State Electrotechnical University, Saint-Petersburg, Russia
[5]Center for Cognitive Science, Kaiserslautern University of Technology, Germany

*To whom correspondence should be addressed; E-mail: nickjarman89@gmail.com

Supplementary Information: Figures

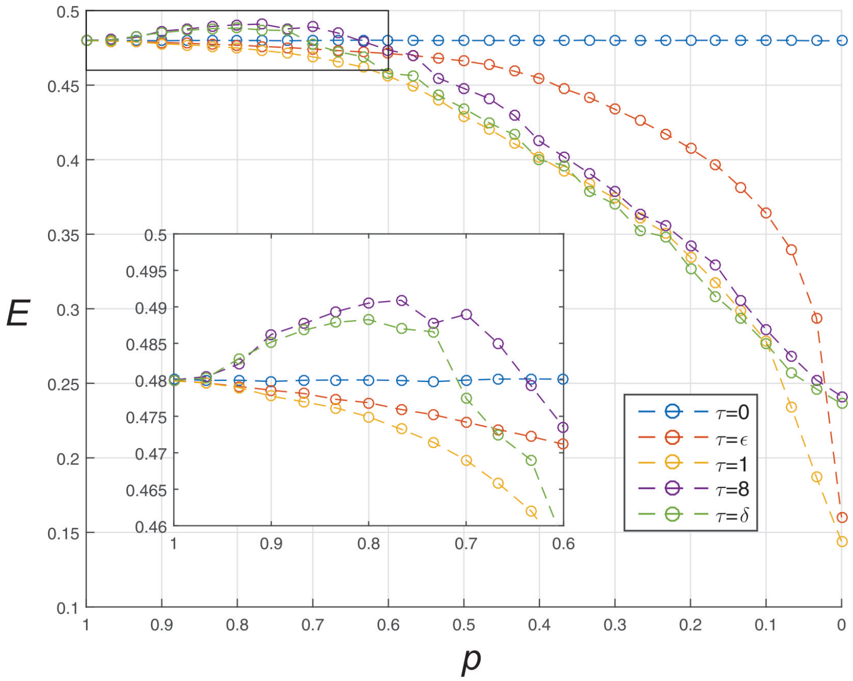

Figure S1: Depicts the global efficiency E as a function of decreasing random rewiring probability $p \in \{0, 1/30, \ldots, 29/30, 1\}$: Coloured lines indicate values of heat kernel parameter $\tau \in \{0, 10^{-15} = \epsilon, 1, 8, 10^{15} = \delta\}$.

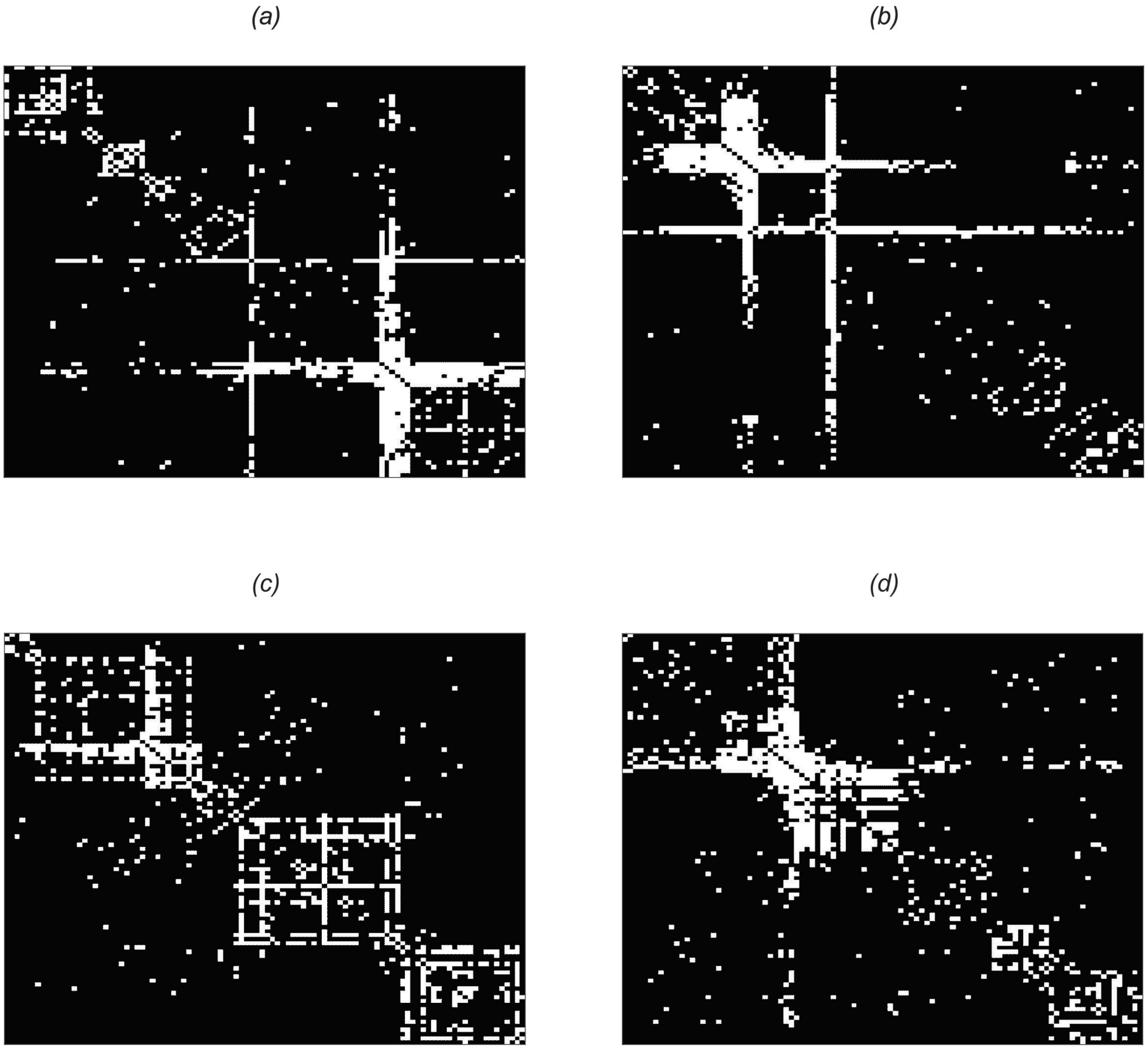

Figure S2: **a-d**: Four independently evolved networks, randomly selected, where in each the pair $(\tau, p) = (5, 0.522)$. Rows and columns of adjacency matrices have been permuted to visualise the modules, in accordance with *(1)*.

Supplementary Information: Adaptive rewiring algorithm using MATLAB

```
n=100; % number of vertices
m=round(2*log(n)*(n-1)); % number of edges
tau=1; % time parameter of heat kernel detmining diffusion rate
p=0.3; % probability of randomly rewiring
k=4*m; % number of edge rewirings

% Generate initial random (symmetric) adjacency matrix
inds=find(triu(ones(n)-eye(n)));
randind=inds(randperm(2\n*(n-1),2\m));
A=zeros(n); A(randind)=1; A=A+A';

% predefined for saving computational cost
I=eye(n); logI = logical(I); IND=1:n;

for edge_rewire = 1:k
    deg=sum(A,2); % vector of vertex degrees
    % select a vertex uniformly randomly such that it has both
        nonzero degree and not fully connected to rest of
        network
    v = find(logical(deg>0 & deg<(n-1))); % vertex v
    v = v(randi(length(v)));

    % randomly rewire with probability p. determine vertices
        u_1 and u_2
    if rand >= p % rewire by network diffusion
        % calculate the graph exponential heat kernel, h(t),
            for t=tau
        deg(~deg)=1; deginv=1./sqrt(deg);
        L = I - bsxfun(@times,bsxfun(@times,A,deginv),deginv');
        h=expm(-tau*L);

        ind=IND; ind(v)=[]; % prevent self-coupling
        [~,u_1] = max(A(:,v)./h(:,v));
        [~,u_2] = max(~A(ind,v).*h(ind,v));
        u_2=ind(u_2);
    else % rewire randomly
        u_2=find(~A(:,v)); u_2(u_2==v)=[]; % prevent self-
```

```
            coupling
        u_2=u_2(randi(length(u_2)));
        u_1=find(A(:,v)); u_1=u_1(randi(length(u_1)));
    end
    % edges are rewired
    A(u_2,v)=1; A(u_1,v)=0;
    A(v,u_2)=1; A(v,u_1)=0;

    % randomly permute adjacency matrix to eliminate any
       ordering of vertices
    rnd=randperm(n);
    A=A(rnd,rnd);

end
```

References

1. Rubinov, M. & Sporns, O. Complex network measures of brain connectivity: Uses and interpretations. *NeuroImage* **52,** (3):1059-1069 (2010).
